# Family tree and ancestry inference: is there a need for a ‘generational’ consent?

**DOI:** 10.1186/s12910-015-0080-2

**Published:** 2015-12-09

**Authors:** Susan E. Wallace, Elli G. Gourna, Viktoriya Nikolova, Nuala A. Sheehan

**Affiliations:** Department of Health Sciences, University of Leicester, Leicester, LE1 7RH UK; Department of Genetics, University of Leicester, Leicester, LE1 7RH UK

## Abstract

**Background:**

Genealogical research and ancestry testing are popular recreational activities but little is known about the impact of the use of these services on clients’ biological and social families. Ancestry databases are being enriched with self-reported data and data from deoxyribonucleic acid (DNA) analyses, but also are being linked to other direct-to-consumer genetic testing and research databases. As both family history data and DNA can provide information on more than just the individual, we asked whether companies, as a part of the consent process, were informing clients, and through them clients’ relatives, of the potential implications of the use and linkage of their personal data.

**Methods:**

We used content analysis to analyse publically-available consent and informational materials provided to potential clients of ancestry and direct-to-consumer genetic testing companies to determine what consent is required, what risks associated with participation were highlighted, and whether the consent or notification of third parties was suggested or required.

**Results:**

We identified four categories of companies providing: 1) services based only on self-reported data, such as personal or family history; 2) services based only on DNA provided by the client; 3) services using both; and 4) services using both that also have a research component. The amount of information provided on the potential issues varied significantly across the categories of companies. ‘Traditional’ ancestry companies showed the greatest awareness of the implications for family members, while companies only asking for DNA focused solely on the client. While in some cases companies included text recommending clients inform their relatives, showing they recognised the issues, often it was located within lengthy terms and conditions or privacy statements that may not be read by potential clients.

**Conclusions:**

We recommend that companies should make it clearer that clients should inform third parties about their plans to participate, that third parties’ data will be provided to companies, and that that data will be linked to other databases, thus raising privacy and issues on use of data. We also suggest investigating whether a ‘generational consent’ should be created that would include more than just the individual in decisions about participating in genetic investigations.

## Background

Genealogical research, or the tracing of ethnic origin and ancestry, although a well-established process for scientific and demographic research, has also become increasingly popular as a recreational activity in recent years. Traditionally, public records including birth and marriage certificates, census statements, interviews and immigration data were the main sources of information for making ancestral links. Companies have been created to assist individuals to ascertain their genetic heritage and fill out family trees, or pedigrees. More recently, technological advances and significantly lower analysis costs have resulted in a number of companies incorporating deoxyribonucleic acid (DNA) analysis further enabling people to seek out relatives. Moreover, direct-to-consumer (DTC) genetic testing services now include ancestry information as one of the benefits of joining. This merging of genealogical research with genetic testing is becoming more commonplace [1]. Genealogical data are routinely being added to biobanks to enrich the resource. Despite the fact that genetic association studies are typically carried out on unrelated individuals, pedigrees have recently been recommended as an ideal design for finding rare genes, for controlling for population stratification effects and for deep sequencing of affected family members [2–4]. In the light of recent methodological developments, pedigrees can now be actively sought in large datasets of supposedly unrelated individuals and can, in principle, be reconstructed from anonymised population cohort study genetic marker data [5–9]. Genealogical research, both for scientific and recreational purposes, is joining the ‘big data’ revolution.

Yet the impact on relatives of a decision by one individual to obtain, and perhaps share, genetic information has not been fully explored. Much literature has been written on best practices in sharing genetic and ancestry knowledge within certain specialties such as clinical genetics and genetic counselling [10, 11]. Revealing misattributed paternity has long been a concern in sharing pedigree information, but additional issues are raised by ancestry testing. The American Society of Human Genetics in their 2008 statement on ancestry testing, notes that it is an inexact science and assumptions can be made based on subjective data, for example, “…imply [ing] clear-cut connections between DNA and specific regions and ethnic groups…”[12]. If the connection is not welcome or at odds with existing beliefs, such implications can cause distress or disbelief. The ASHG notes that, “[t]he occurrence of or potential for emotional distress in people or groups following receipt of conflicting information about their ancestry has been documented, but still needs more research [12].

The rise in popularity of recreational genomics, seen through television programmes and company advertisements, shows that people are eager to seek out and share their ancestry data. But, genetic information implicates not only the individual but their biological relatives and social family, and third parties to ancestry investigations have received limited consideration. A 2015 statement from the Genetic Genealogy Committee, an independent group of genealogists clearly recognises the implications for third parties but makes no recommendation for ensuring such parties are informed of the intent to participate in one of these companies [13]. The ease with which genealogical and other personal data from the client, and by extension from their relatives, can be shared, linked and used, raises issues of who gives consent to provide that data and how well all parties are aware of the implications of participation.

Informed consent is an individualistic process designed to allow a capable individual, with sufficient information and time, to make a decision regarding participation. One could theorise that because genetic information implicates others beyond the individual, the information on which the consent is based should include details regarding the potential implications of participation on families and relatives. Hudson et al. and the ASHG have recommended that, “Companies offering DTC testing should clearly disclose all risks associated with testing, including psychological risks and risks to family members” [14]. Using our knowledge of best practice in the creation of consent forms, through previous work including creating consent templates [15], we examined privacy and data management procedures used by companies offering ancestry testing and estimation to determine whether the needs and wishes of family members (biological relatives, in particular) are taken into account when an individual chooses to use these companies’ services and when (s)he opts to share the findings from the performed analyses with other users on online databases.

## Methods

We first conducted a preliminary search using the databases PubMed, Scopus and Web of Knowledge looking for papers discussing recreational and genetic ancestry. At the same time we searched using the search engine Google for companies providing recreational genealogy services, (genetic) ancestry testing and conducting related research. We used the following key words (and combinations of keywords) to focus our search specifically in the area of genealogy and ancestry testing: genealogy, genealogy service, recreational genealogy, genetic genealogy, family history, family tree, ancestry, combined with the words company OR service OR organisation OR provide OR business. We excluded key words such as DNA testing as they moved the search into the domain of clinical testing which was outside the focus of this paper.

The search revealed numerous companies offering a wide range of genealogy and genetic services and several types of ancestry tests. To limit our results we used the following inclusion criteria: a) web-page in English; b) providing commercial services; c) located in the United Kingdom or United States; and d) a primary focus on recreational ancestry using data and/or DNA samples provided by an individual (hereafter ‘client’). We excluded companies or laboratories that primarily offer a specific type of test other than ancestry (e.g. paternity test, immigration or forensic test) and websites that only promote such services. Because many companies had multiple locations and provided services online, we abandoned the location criteria. To ensure that no companies previously studied were overlooked, the list of genetic genealogy companies produced by Royal et al. [16] was also reviewed. Twenty-one companies, including subsidiary companies, fulfilled our criteria and were included in the analysis (Table 1).

**Table 1:** Characteristics of companies included in the analysis

Companies	Ancestry services based on self-reported data	Ancestry services based on a DNA sample	Ancestry services based on self-reported data and a DNA sample	Offers a Research Component in addition to other services
23andMe (23andMe Research)			**√**	**√**
African Ancestry		**√**		
African DNA		**√**		
Ancestry by DNA		**√**		
Ancestry^a^	**√**			
AncestryDNA^a^ (Ancestry Human Diversity Project)			**√**	**√**
AncestryHealth^a^ (Ancestry Human Diversity Project)	**√**			
Archives^b^	**√**			**√**
Archives^b^	**√**			
DNA Testing Systems		**√**		
DNA Tribes		**√**		
Family Search	**√**			
Family Tree DNA (various projects)			**√**	
FamilyLink^b,c^	**√**			
Find My Past	**√**			
Genes Reunited	**√**			
Geni^b^	**√**			
MyHeritage^b,c^	**√**			
Oxford Ancestors		**√**		
Roots for Real		**√**		
The Genealogist^b^	**√**			
World Vital Records^b,c^	**√**			

^a^AncestryDNA and AncestryHealth are subsidiaries of Ancestry

^b^These companies outsource DNA testing to other companies (FamilyLink, GENI and The Genealogist, World Vital Records use FTDNA; Archives use Ancestry; and MyHeritage uses FTDNA or 23andMe)

^c^FamilyLink, GENI and World Vital Records are subsidiaries of MyHeritage

We conducted a directed content analysis [17] to validate our theory that for consent to be informed, individuals should be notified of the potential implications of participation on their family and relatives. We collected publically available consent policies, privacy policies and terms of conditions (hereafter T&C, or terms of use) documents and highlighted what information is given to the potential client, what consent is required, what risks associated with testing were mentioned, and whether obtaining the consent of or the need to notify third parties was mentioned. Homepages; “About us” pages; and Frequently Asked Questions (hereafter FAQ) pages were also searched. All documents were imported and coded using NVivo 10 to facilitate the analysis. Initial codes were generated from the research questions that guided but did not constrain the analysis. Codes were developed and iteratively revised. They included (but were not limited to) consent for the individual, alternative forms of agreement, consent for third parties, proof of consent and family considerations. In the first stage of the analysis, companies were placed in categories based on the services provided. Next, detailed charts were produced that included coded sections of text for each group. Similarities and differences in text were compared; we also highlighted where text was included or when it was missing. Based on these tables we were able to judge whether there was overt discussion of the implications for others as a result of consenting to participate.

## Results

We identified four categories of companies offering recreational genealogy services (Table 1): 1) companies that provide genealogy services based only on self-reported data, such as personal or family history; 2) companies that provide genealogy services based primarily on a DNA sample provided by the client; 3) companies that provide genealogy services using both self-reported data and that from the DNA sample; and 4) companies that also have a research component.

### Group 1: companies that provide ancestry services based on self-reported data

Ten companies were included in the first group: Ancestry, AncestryHealth, Archives, FamilyLink, Family Search, Find My Past, Genes Reunited, Geni, MyHeritage, The Genealogist, and World Vital Records. These companies provide what might be called ‘traditional’ family history services. They help users discover their distant relatives through access to census records, marriage and death records, military records, etc. Clients provide personal information (name, gender, year of birth, address etc.); personal information for other people (e.g. names and birthdays of family members); and dates and places of events (e.g. birth, death, marriage, divorce, immigration, etc.). Clients may keep their family histories private if they wish. Alternatively, they may choose to make their histories openly available enabling other service users to search these histories for common relatives. AncestryHealth, which focuses on health history, suggests clients may wish to share their AncestryHealth records with their physicians [18].

All but one of the companies (The Genealogist) mention in their Privacy Policy or T&C document that consent is implied by the use of the website or the purchase of their products. For example, “By accepting these Terms and registering as a member of the Genes Reunited Service, you give your consent for your personal data to be stored and processed in accordance with our Privacy Policy” [19]. The majority of the companies (eight of ten) also recognise that potential complications may arise from providing and sharing genealogical information that relates to individuals other than the client. This is evidenced by the frequent presence of statements emphasising that it is the responsibility of the client to obtain consent from living family members prior to sharing information about them on the website and to make the implications of that consent clear. Moreover, some companies also insist that their clients should obtain the consent of third parties and be able to provide the company with the necessary documentation, if requested (Table 2).

**Table 2:** Quotes from company privacy policy or T&C documents referring to the need to obtain consent from family members

Company	Quote from privacy policy/T&C document
Ancestry	You should obtain the consent from the living persons about whom you want to post personal information on the Websites, or, if the person is under the age of 18, the consent of their parent or guardian [48].
AncestryHealth	Before providing health and lifestyle information about others, you represent that you have obtained the consent from them, or, if the person is under the age of 18, the consent of their parent or guardian [49].
Archives	[…][Y]ou should only submit or share User Provided Content that belongs to you (or where you have obtained all necessary permissions or consents) and that will not violate the rights of others [50].
Family Search	When providing personal information about anyone other than yourself, you must first obtain the other person’s informed consent if his or her consent is legally required [according to local law] [51].
FindMyPast	You should always seek permission from people who are living before you make their personal information available in your tree, or anywhere else online. […] We reserve the right (at our own discretion) to remove any personal data which you have included in your family tree about people who are living if we are alerted to the fact that this personal data was used without that person’s permission [52].
GenesReunited	You promise that you are the original owner of any Submission you make or you have the necessary licences, rights, consents, and permissions to authorise us to use your Submission. In particular, you promise that you have obtained the permission of all of the living people featured or referred to in the Submission (and if they are under 18 their parents’ or guardians’ permission as well).[…] You agree to give us evidence of all such licences, rights, consents, and permissions if so requested by us [19].
Geni	[I]s prohibited to […] posting any third party’s (including without limitation any family member’s) information on the Geni Services without permission [53].
MyHeritage	In addition you should also make sure that information or material you wish to place on the Website about anyone living is only posted with their prior knowledge and consent. If the person is under the legal age to enter into agreements (typically 18 years old), you represent that you have obtained the consent of the parent or guardian of the person under the legal age […]. In all cases, you must make the implications of the consent clear to the person (or, if applicable, to the parent/guardian) [54].

As noted by FindmyPast, controls are included to allow clients to decide which pieces of information can be shared openly.

On some parts of the website, you can publish things (including your family tree), make comments or participate on forums. If you do, you must not: publish something that you do not own the copyright in (or have permission to publish it from the copyright owner); […]; or share the personal information of living people without their permission. You’re responsible for managing content you create, including using privacy settings we make available [20].

Six of these companies (Archives, FamilyLink, Geni, My Heritage, The Genealogist and World Vital Records) collaborate with other companies that offer DNA tests to enrich the ancestry information that can be inferred. In these cases, a separate link is provided that directs the client to the privacy policy document of the company offering the DNA test. More specifically, MyHeritage offers its clients the option of using either 23andMe or FamilyTreeDNA for the DNA test. FamilyLink, Geni, The Genealogist and World Vital Records uses FamilyTreeDNA, while Archives collaborates with AncestryDNA. These are discussed below (Group 3).

### Group 2: companies that provide ancestry services based on a DNA sample

Seven companies (African Ancestry, African DNA, Ancestry by DNA, DNA Testing Systems, DNA Tribes, Oxford Ancestors and Roots for Real) provide ancestry services based mainly on the analysis of a DNA sample provided by the client. These offer information about the individual’s ancestral origins, such as the percentage of their ancestors who were of African, European and Native American ancestry. Three of the companies (AfricanAncestry, DNAConsultants and OxfordAncestors) have a “consent by use” statement. For example, Oxford Ancestors states that, “By placing an order you signify that you freely and specifically consent to the collection and processing of any personal data you provide” [21]. African Ancestry asks clients to confirm that they have the right to submit samples from others: “By submitting a sample for analysis, the Customer warrants that it has the right to take and submit the sample and that it does so either as owner of all samples involved or with full authority of the owner of all such samples” [22]. Concerns over ownership of samples as cited by these companies could be interpreted to reflect the fact that in some countries, such as the UK, legislation is in place to protect against the collection and use of DNA samples without the explicit consent of the person from whom it was taken [23]. The remaining four companies do not have a consent by use statement and no specific mention is made by any of the companies about the ethical implications for third parties, such as possible adverse psychological reactions to genetic ancestry data [12].

### Group 3: companies offering ancestry services based on self-reported data and a DNA sample

Three companies (23andMe, AncestryDNA and Family Tree DNA) collect a wide range of information (name; address; health-related information; personal traits; and family history) and DNA samples. They use them to provide information about one’s deep ancestral origin as well as a list of potential genetic matches (potential relatives) from the company’s ever expanding database. In addition, 23andMe, through only their United Kingdom, Canadian and European branches, also offer health-related information and specifically, using variants or markers, information about genetic risk factors, inherited conditions, pharmacogenetics and other traits (e.g. earwax type). 23andMe was suspended from providing health-related data to their clients in the United States in 2013; however, they are taking steps to return to their previous position by recently receiving marketing approval from the US Food and Drug Administration for a carrier test for Bloom Syndrome [24].

As previously mentioned, these companies provide services to clients referred through other ancestry companies. These companies may also outsource the actual DNA analyses to specific (usually Clinical Laboratory Improvement Amendments (CLIA) certified) laboratories. 23andMe uses LabCorp for the genotyping services while Family Tree DNA uses the Genomics Research Center run by GenebyGene. As with other companies discussed, consent is deemed to be given when services are purchased. “By using the AncestryDNA Website and the Services you consent to the collection, use, storage and disclosure of your Personal Information by AncestryDNA in accordance with this Privacy Statement” [25].

These companies however are more specific when detailing the services to which one is consenting. 23andMe, for example, is very explicit about the circumstances under which the information provided is going to be used.

By agreeing to our Privacy Statement and Terms of Service, you consent to sensitive information, such as information about your health, Genetic Information, and Self-Reported Information such as racial and ethnic origin and sexual orientation (where you provide it) being used by us to: analyze and provide you with our Services; analyze and provide you with information about your ancestry; determine whether you would be suitable to take part in surveys, polls or questionnaires that we are conducting; and monitor and improve existing products or services that we offer or develop new products and service [26].

Furthermore, 23andMe and AncestryDNA demonstrate concern for third parties by asking their clients to obtain consent from other people before using their information. AncestryDNA states that,

You should obtain the consent from the living persons about which you want to post personal information on the Websites, or, if the person is under the age of 18, the consent of their parent or guardian [27].

23andMe cautions, “Where you are disclosing information about a family member, you should make sure that you have permission from the family member to do so”[24]. Interestingly, 23andMe recently added a new page to its website specifically focusing on “Family Considerations”.

Looking at your genetic data might uncover information that some people find surprising. This information can be relatively benign and even amusing. At other times, the information you learn can have profound implications for both you and your family [28].

In that document they cover issues relating to families (revealing information about new biological relatives or misattributed paternity); ancestry; health (increased risk of a particular condition) and relationships. They specifically mention:

Because genetic information is hereditary, knowing something about your genetics also tells you something about those closely related to you. Your family may or may not want to know this information as well, and relationships with others can be affected by learning about your DNA. Everyone has different tolerances and preferences for learning information. You might be surprised by a family member who would prefer not to know something you feel is important to share. At other times, you may learn something about yourself, your family, your ancestry, or health-related associations with your genotype that you would prefer to keep private. You may find yourself having to weigh sharing such information with other family members against your own desire for privacy - or their desire not to know [28].

### Group 4: companies that also offer a research component

The companies in category 4 (23andMe, AncestryDNA, AncestryHealth, and FamilyTreeDNA) have an open research component where clients can give consent for the use of the genealogical data and DNA samples to be used for a variety of research purposes. These studies range from more traditional genealogical research with non-profit groups [29] to biomedical research using genetic data.

Family Tree DNA offers the opportunity to participate in a variety of volunteer-lead projects and to combine personal DNA data with that from others to explore, for example, the history of particular surnames or geographical locations. Clients are informed about the necessary broad and explicit consent required depending on the circumstances. General consent is needed to participate in any GenebyGene services, while explicit consent will be asked for participating in specific research activities, which may involve allowing access to the anonymised data or to their personal, non-anonymised data, depending on the study. Their research partners may include, “…commercial or non-profit organizations that conduct or support population genetic studies, scientific/medical research, or the development of drugs or devices to diagnose, predict, or treat health conditions” [30].

The Ancestry Human Diversity Project is run by Ancestry and can be joined through either AncestryDNA or AncestryHealth. This project also seeks, “to better understand, among other things, human evolution and migration, population genetics, population health issues, ethnographic diversity and boundaries, genealogy, and the history of our species” [31]. Both companies provide publically available consent forms [32, 33] describing the information that might be required (e.g. genealogical and genetic information) and ask potential clients to sign it in addition to accepting the T&C of the company. Although a wide range of information is requested, including pedigree and family history data, no specific concerns are expressed regarding third party interests: they only acknowledge that test results might reveal information about others. For example, AncestryDNA states the following:

Your test results may reveal information about you or your biological family (blood relatives), but there are no physical risks for providing a saliva sample and having your sample and Information used in this Project [32].

At this time there is no formal link between AncestryDNA and AncestryHealth. However, industry observers believe that it is only a matter of time before these two subsidiaries link their family history, self-reported and DNA data to enable Ancestry.com to provide health-related information to clients [34], in a similar way to 23andMe as described above.

23andMe Research shows the company’s decision to move decisively into biomedical research. It works with patient communities, academic researchers and pharmaceutical companies, and publishes its results in peer-reviewed journals. All research activities are overseen by an independent institutional review board. Due to its large client population and access to associated data, it claims to be able to produce useful research results in a shorter time than other more traditional biomedical research projects [35]. Their research consent states that genetic and self-reported information will be collected *including* information submitted prior to giving consent [36]. With regard to the possible risks associated with such consent, their research consent document only mentions that, “[s]ome survey questions may make you or your family members uncomfortable” [36]. While this acknowledges that the use of this information might affect family members, it does not require family members to consent, and it does not mention that family members should be informed about the client’s intention to join a research project. The Family Considerations page, provided by 23andMe, is not repeated for 23andMe Research.

## Discussion

Our analysis showed that the amount of text discussing the need to take third parties into consideration, whether biologically related or not, varied substantially across the activities we examined. Companies solely offering more ‘traditional’ ancestry services, without DNA analyses, more clearly highlight the issues. They acknowledge that ancestry information is shared across families and that their consent should be sought. However, when DNA analysis is included or is the sole means of participation, there is a shift to language focusing on the protection of the use of personal or sensitive information of the client and ownership of DNA samples, with little language about third parties. Once activities move from ‘recreational’ to ‘research,’ such as with the four companies offering a research component to clients, the language reflects existing regulations and norms, such as the need for independent ethics oversight, but not implications for third parties. With the exception of 23andMe’s *Family Considerations* text, little focus is placed on the implications for the use and spread of data beyond the individual.

These differences appear to indicate that family history data and DNA are thought of in different ways. Attitudes towards family history data reflect its name – people recognise it as involving more than just the individual. Attitudes towards the brokering of DNA, on the other hand, can be seen in two different ways. Family considerations may not be discussed because of a lack of understanding of its familial nature. Or, it may be because companies have chosen a goods-for-service paradigm which is commonly used in business and protected through the law. This confirms our finding that companies see the client as the single decision-maker on whether their information and any family history, pedigree or personal data of others with whom they are related or associated should be shared on the company’s database or across companies. This is not surprising as their ‘contract’ is with the purchaser of their services. They may highlight the issue, such as with 23andMe, but again only with the client. Any language is mainly placed in privacy policies or terms of use agreements, documents that may not be readily obvious, or even of interest, to the client. Such a model places the onus of knowing the implications and communicating those to the client.

Allowing clients to self-regulate their family’s privacy may no longer be sufficient. The level of understanding that clients possess regarding genealogy information is questionable [37, 38] and it is not known how many actually read the terms and conditions or privacy statements provided. Moreover, while it is obvious to most people that dates and records of family members might not be personal data and could affect others, it is not clear that a saliva sample is viewed the same way. Much has been written about fallibility of anonymisation. Given expertise, resources and will, it is possible to re-identify individuals from anonymised family history data [39] suggesting that procedures such as name removal and encoding are not sufficient to protect against privacy breaches. Another concern is whether clients or third parties actually know that their data may move to jurisdictions with differing data protection regimes. Linkage is another potential concern. Family Tree DNA offers to their clients the possibility to transfer in their raw data from the databases 23andMe or AncestryDNA to find new possible matches in their database. AncestryDNA and AncestryHealth are expected to link their extensive datasets. Genealogical databases are contributing to scientific research, such as through Familinx which combines and links pedigrees contributed by My Heritage clients and others for scientific research [40]. But they have also been used to show that, together with other public on-line resources, “…full identities of personal genomes can be exposed via surname inference” [41]. Do relatives understand the possibilities? Is one person’s consent enough or is something more needed?

## Conclusions

Traditional informed consent reflects individualistic decision-making. We argue that it is time to think of consent in broader terms, as a discussion that, when involving genetic information, goes beyond the individual and asks all parties to think about and involve the broader family and biological relatives. In the context of this study, we suggest companies notify potential clients of the implications for third parties and ask them to inform those third parties of plans to participate. While it would continue to be the responsibility of the client to decide who should be notified, we suggest companies should not ignore the possible ramifications for their clients. At a minimum this could be additional text clearly displayed in a location where it will be seen and written in accessible language, and a line on the consent form that when ticked indicates agreement. We recognise that ensuring clients are aware of this information is difficult, and we invite further discussions with the companies studied to determine how such provisions might be put into place and successfully followed.

We also suggest that these broader discussions could be seen as the basis for creating a ‘generational consent’ model. For this we can take the lead from those involved in traditional forms of family association studies and genetic counselling – begin with the individual and let them take you to the third parties to continue the discussion. We also should take advantage of the enthusiasm shown by people becoming involved in ancestry, genealogy and genetic investigations to open and expand the dialogue. There will be instances of harm, such as misattributed paternity, and we must continue to investigate greater protections for personal data. There will be legal questions and debates regarding the ownership of genetic data [42–44]. But re-identification is happening and ancestry data is moving into human subjects research and being used to inform healthcare decisions. More openness and discussion is needed around the collection, linkage and use of pedigree data. If generational consent is seen a way forward, the intricacies of such a process will need considerable thought and discussion. Middleton et al., in their study of the attitudes toward the return of incidental results from sequencing research, note that, “…genetic health professionals and genomic researchers agree that ancestry is not a category of data for which it is appropriate to search for and to share,” yet the members of the public they interviewed showed more interest in this information [45]. More research is needed to find out what the ‘generations’ around the individual think about the sharing and use of ancestry data and the implications of learning information that may not be wanted [46]. According to ‘Amy’, as quoted on the GenesReunited website, “I found my sister and mother after 1 h of logging on to this site after 53 years of not knowing where they were” [47]. But there is no word on whether Amy’s mother and sister were happy to be ‘found.’

## Abbreviations

CLIA: Clinical laboratory improvement amendments
DNA: Deoxyribonucleic acid
DTC: Direct-to-consumer
FAQ: Frequently asked questions
T&C: Terms and conditions
